# Cell Surface-Specific N-Glycan Profiling in Breast Cancer

**DOI:** 10.1371/journal.pone.0072704

**Published:** 2013-08-23

**Authors:** Xia Liu, Huan Nie, Yubao Zhang, Yuanfei Yao, Alaiyi Maitikabili, Youpeng Qu, Shuliang Shi, Cuiying Chen, Yu Li

**Affiliations:** 1 School of Life Science and Technology, Harbin Institute of Technology, Harbin, Heilongjiang, China; 2 Tumor Hospital of Harbin Medical University, Harbin, Heilongjiang, China; 3 Department for Molecular Biomedical Research, VIB, Ghent, Belgium; 4 Department of Biomedical Molecular Biology, Ghent University, Ghent, Belgium; The University of Hong Kong, China

## Abstract

Aberrant changes in specific glycans have been shown to be associated with immunosurveillance, tumorigenesis, tumor progression and metastasis. In this study, the N-glycan profiling of membrane proteins from human breast cancer cell lines and tissues was detected using modified DNA sequencer-assisted fluorophore-assisted carbohydrate electrophoresis (DSA-FACE). The N-glycan profiles of membrane proteins were analyzed from 7 breast cancer cell lines and MCF 10A, as well as from 100 pairs of breast cancer and corresponding adjacent tissues. The results showed that, compared with the matched adjacent normal tissue samples, two biantennary N-glycans (NA2 and NA2FB) were significantly decreased (*p* <0.0001) in the breast cancer tissue samples, while the triantennary glycan (NA3FB) and a high-mannose glycan (M8) were dramatically increased (*p* = 0.001 and *p* <0.0001, respectively). Moreover, the alterations in these specific N-glycans occurred through the oncogenesis and progression of breast cancer. These results suggested that the modified method based on DSA-FACE is a high-throughput detection technology that is suited for analyzing cell surface N-glycans. These cell surface-specific N-glycans may be helpful in recognizing the mechanisms of tumor cell immunologic escape and could be potential targets for new breast cancer drugs.

## Introduction

Breast cancer is the commonest cancer and the leading cause of cancer death in women. It is well known that early detection and diagnosis could dramatically improve breast cancer patient 5-year survival rates [1]. At present, several approaches, such as mammography, have been applied in breast cancer diagnosis. However, these common diagnostic approaches show unsatisfactory performance in sensitivity and specificity. Therefore, there is an urgent need to find effective biomarkers for breast cancer diagnosis. Over the past few years, several large-scale proteomic studies have been carried out to characterize the proteome of breast cancer to identify biomarkers [2–4]. However, this strategy leads to complex data sets, which make it hard to predict sensitive and specific targeted proteins in breast cancer.

Glycomics has recently garnered much attention. Glycosylation is the most complex post-translational modification step in the biosynthesis process of proteins. A particular type of N-glycans is closely associated with the biological functions of cells [5,6], such as the growth, differentiation, adhesion and metastasis. The sugar chains of glycoproteins are important for maintaining the ordered “social behavior” of differentiated cells in multicellular organisms. Furthermore, alterations of the sugar chains also contribute to the molecular mechanisms of abnormalities such as inflammation, invasion and metastasis of tumor cells [7]. Actually, some studies have shown that several specific types of glycan epitopes are closely associated with many types of cancer [8,9]. However, there is still much to be understood since the research on glycomics still falls behind that of genomics and proteomics due to the complexity of glycosylation at the molecular level and the lack of powerful analytical tools.

Recently, matrix-assisted laser desorption/ionization-time-of-flight mass (MALDI-TOF MS), high performance liquid chromatography (HPLC), lectin microarrays and DNA sequencer-assisted fluorophore-assisted carbohydrate electrophoresis (DSA-FACE) are the main methods for N-glycome profiling and structural analysis, which constitute the core of research in glycomics [10]. Among them, DSA-FACE has been shown to be a robust, high-throughput, exceedingly sensitive, and reliable quantitative method [11]. In DSA-FACE, 96-well plate-based sample preparation allows high-throughput automatic analysis using multi-capillary separation and facilitates exoglycosidase sequencing without the removal of treated enzymes. This technique has been successfully applied for screening serum glycoprotein biomarkers from healthy people [12–15], patients suffering various diseases [16–21] and cancer patients as well [22–27]. Our group also previously investigated the relationship between human serum N-glycan profiles and age and gender of healthy humans using DSA-FACE [15]. However, to the best of our knowledge, the application of this technique has yet to be demonstrated in the analysis of cell surface-specific N-glycans from cells or tissue specimens.

In this study, we modified the DSA-FACE technique to make it suitable for analyzing the profiles of cell surface-specific N-glycans from human breast cancer cell lines and tissues. Using this modified method, we report the N-glycan profiles of 8 cell lines and 100 pairs of matched normal and tumor tissues from breast cancer patients. Our results indicate that the DSA-FACE technique was also capable of detecting alterations in cell surface-specific N-glycans from breast cancer cell lines and tissues. The N-glycan profiles showed that peaks B1 (NA2) and B4 (NA2FB) were significantly decreased, while peaks B2 (mannose 8, M8) and B5 (NA3FB) were significantly increased in tumor samples in comparison with those of the control group. The increased N-glycans could be regarded as potential targets for drugs in tumor therapy, and the decreased N-glycans would be beneficial in studying the relationship between glycans and the immune evasion of cancer cells. Meanwhile, knowledge of these specific N-glycans may help in understanding the mechanisms of tumorigenesis, progression and metastasis of breast cancer.

## Materials and Methods

### Specimens and cell culture

The Tumor Hospital of Harbin Medical University provided 100 pairs of matched adjacent normal and tumor specimens from patients with histologically proven breast cancer, which were frozen at -196^o^C before analysis. Board certified clinical oncologists and pathologists carried out all clinical and histological analysis of the biopsies. The stage was classified according to the TNM criteria (2002) [28]. The use of human breast tissues in this study was approved by the Ethical Committee of the Tumor Hospital of Harbin Medical University, and was in accordance with the ethical standards laid down in the 1964 Declaration of Helsinki for research involving human subjects. The written informed consents of all participating subjects were obtained before commencement of the study.

Seven breast cancer cell lines SK-BR-3, MCF-7 and MDA-MB-231 from breast adenocancer, T-47D and ZR-75-30 from breast ductal cancer, Bcap37 from squamous cell cancer, Hs-578T from mammary gland cancer and MCF 10A as a non-tumorigenic epithelial cell line were used in this work. All cell lines were purchased from American Type Culture Collection (ATCC) with the exception of Bcap37, which was purchased from the Shanghai Institute Cell Bank. All cell lines were cultured according to supplier’s guidelines. For glycan analysis, cells were detached using a cell scraper, washed with PBS, pelleted and frozen at -80^o^C until analysis.

### Preparation of membrane proteins

Membrane proteins from cell or tissue specimens were extracted with a slightly modified detergent-based protein extraction method adopted by Clark et al. [29]. In brief, 1×10^7^ cells or 200 mg ground tissue specimens were collected and 1 mL ice-cold lysis buffer containing 100 mM Tris-HCl (pH 7.5), 1 mM EDTA, 2% Triton X-114 and 1% protease inhibitor mixture (10 mg/mL PMSF, 2 mg/mL Leupeptin, 200 µg/mL Pepstatin, 2 mg/mL Aprotinin) was added. The cell lysate was centrifuged at 13,000 rpm for 20 min at 4^o^C to remove insoluble cell debris and then an equal volume of sucrose cushion containing 6% sucrose, 150 mM NaCl, 0.06% Triton X-114 and 10 mM Tris-HCl was added to the supernatant, followed by incubation in a 37^o^C water bath for 5 min. After centrifugation, the supernatant was discarded and 9-fold volume of acetone precooled at -20^o^C was added to the detergent phase to precipitate protein. Finally, after overnight incubation at -20^o^C, the acquired membrane protein was collected by centrifugation at 13,000 rpm for 20 min at 4^o^C, dried in a vacuum, resuspended in 50 µL deionized water and stored at -80^o^C.

The concentration of the membrane protein was determined by Lowry assay [30]. Membrane proteins were identified by Western blot using anti-Na^+^/K^+^ ATPase-α1 antibody (Santa, U.S.) as the first antibody, and cytoplasmic proteins were distinguished by anti-γ-tubulin antibody (Santa, U.S.). Signals were developed with an enhanced chemiluminescence Western blotting detection system (ECL kit, GE healthcare).

### Preparation of N-glycans from membrane proteins

The preparation of N-glycans from membrane proteins was composed of PNGase F digestion, GC resin purification, removal of sialic acid, fluorescence labeling and Sephadex G10 purification steps. In order to analyze the N-glycan profiling of cell/tissue specimens, all the parameters involved in above N-glycans preparation protocol which fit for DSA-FACE analysis of serum specimens, especially the quantities of cell/tissue specimens, enzymes and regents, were reset. In addition, a GC resin purification process was introduced in sample preparation to remove remaining polypeptides, detergent, monosaccharides and salts from the digestion mixture containing glycans. The modified protocol for the N-glycans preparation process is as follows:

For the PNGase F (NEB, P0704L) digestion, 6 µL of denaturing buffer containing 5% SDS and 10 mM NH_4_HCO_3_ (pH 8.3) were added to 15 µL of membrane proteins. Then, the acquired suspension was heated at 95^o^C for 10 min, followed by cooling at 4^o^C for 15 min. To release the N-glycans from the proteins, 4 µL of PNGase F solution containing 500 units PNGase F and 3.33% NP-40 were added to each tube followed by incubation at 37^o^C overnight.

The released N-glycans were purified using non-porous graphitized carbon resin (GC resin) from Carbograph [31] with some modifications. Briefly, 40 mg of GC resin were loaded into each well of a 96-well plate and pre-activated by 5 cycles of washing with 200 µL washing buffer containing 80% (v/v) acetonitrile and 0.1% trifluoroacetic acid (TFA) and then with 200 µL deionized water. During this process, the washing buffer and deionized water were removed via centrifuging the plate at 30 g for 5 min (Beckman, Allegra^TM^ X-22 centrifuge). After the addition of PNGase F reaction mixture obtained from last step, each well on the plate was washed 5 times with 200 µL deionized water followed by centrifugation at 200 g for 20 s to remove the remaining salts, monosaccharides and detergent. Finally, N-glycans were sequentially eluted twice with 200 µL elution buffer I (10% acetonitrile in H_2_O), elution buffer II (20% acetonitrile), elution buffer III (25% acetonitrile, 0.05% TFA) and 3 times by 200 µL elution buffer IV (40% acetonitrile, 0.05% TFA). The flow rate of centrifugation was maintained at 0.5-1 mL/min. The elute with N-glycans was collected in a 200 µL EP-tube and dried in a vacuum for further use.

To remove sialic acid, the dried glycans were redissolved in 25 µL deionized water and mixed with a 12 µL neuraminidase (NEB, P0720L) solution containing 30 units of neuraminidase, 3 µL G1 buffer and 8.4 µL H_2_O, and were incubated at 37^o^C overnight.

To label the desialylated N-glycans with 8-amino-1, 3, 6-pyrenetrisulfonic acid (APTS), the samples were dried completely at 65^o^C for about 3 h, and after cooling they were combined with 5 µL of APTS labeling buffer (1:1 of 20 mM APTS in 1.2 M citric acid and 1 M NaCNBH_3_ in DMSO). The tube was sealed and heated for 2 h at 90^o^C and this reaction was stopped by adding 20 µL of water.

Finally, the labeled N-glycans were purified with Sephadex G10 as previously described [11]. In brief, the sample was loaded on the well of a Multiscreen-Durapore membrane-lined 96-well plate (Millipore) with 100 mg/well Sephadex G10 and was eluted by 3 cycles of washing with 20 µL of deionized water by centrifugation at 750 g for 10 s. The collective samples were dried in a vacuum apparatus and were stored at -20^o^C for further analysis.

### Glycan analysis with a DNA sequencer

The resulting APTS-labeled glycans were dissolved in 15 µL deionized water and 10 µL of the solution was dispensed into the wells of a 96-well plate, which was loaded onto an ABI 3130 sequencer equipped with a standard 36 cm capillary array filled with the POP-7 polyacrylamide linear polymer. The running parameters for the sequencer were the same in the previously described protocol [11]. The resultant electropherograms were then analyzed using GeneMapper 4.0 software. Each structure of N-glycan was numerically described by normalizing its height to the sum of the heights of all peaks.

### Structural analysis of the N-glycan pool using exoglycosidase digestion

An appropriate number of APTS-labeled N-glycans, obtained with the procedure described above, were digested overnight with different mixtures of exoglycosidases in 10 mM NH_4_Ac (pH 5.0) at 37^o^C. The enzymes in use included *Streptococcus pneumoniae* β-1, 4-galactosidase, bovine kidney α-1, 6-fucosidase, almond meal α-1, 3/4-fucosidase and *Aspergillus saitoi* α-1, 2-mannosidase. All enzymes were from Prozyme, San Leandro, CA. DSA-FACE was used to analyze the digestion products.

### Statistical analysis

All quantitative variables were presented as mean ± SD. Paired and unpaired *t*-tests were used for comparison of the two tissue groups, and for comparison of more than two cell line groups, a one-way ANOVA was applied. Pearson coefficients of correlation (Spearman coefficients of correlation were calculated for ordinal categorical variables) and their associated probability (p) were used to evaluate the relationship between parameters. All reported *p* values were two-tailed, and *p* values <0.05 were considered statistically significant. Statistical analyses were performed with SPSS10.0 software.

## Results

### Establishment of the method for analyzing N-glycan profiles of membrane proteins from cell and tissue specimens based on the DSA-FACE technique

**Figure 1:**
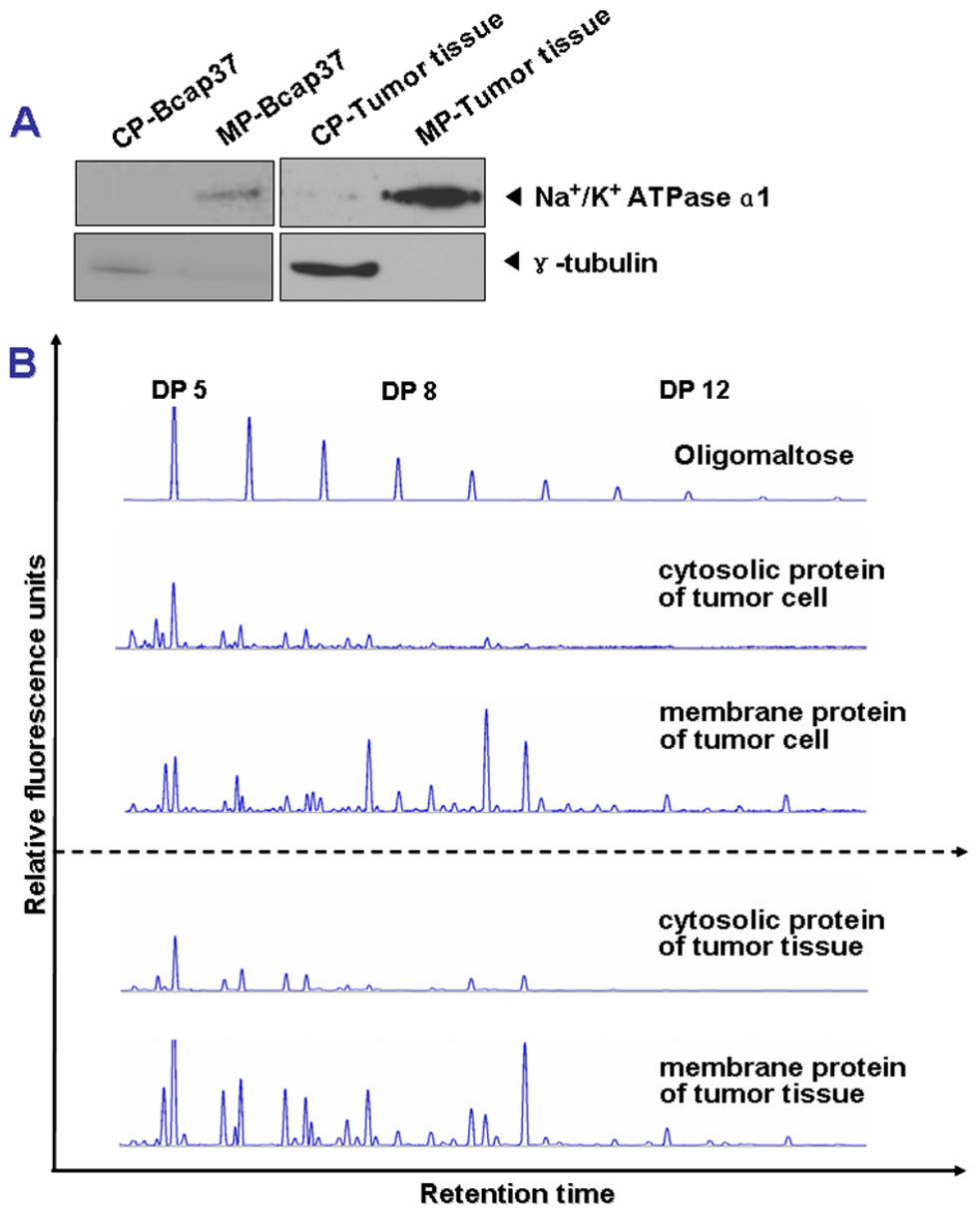
Establishment of the DSA-FACE method for analyzing N-glycan profiling in breast cancer. (A) Equal amounts (20 μg and 100 μg for cell line and tissue specimens, respectively) of cytoplasmic and membrane proteins (CP and MP) from Bcap 37 cells and tumor tissue were determined by Western blotting using γ-tubulin and Na^+^/K^+^ ATPase-α1 antibody, respectively. Na^+^/K^+^ ATPase-α1 (an integral membrane protein) and γ-tubulin (a core component of the centrosome) were chosen as the cytomembrane and cytoplasm markers, respectively. (B) The representative N-glycan profiles of cytosolic and membrane proteins from cell line (upper panel, n=8) and tissue (lower panel, n=200).

As shown in Figure S1, the DSA-FACE technique was modified to analyze the N-glycan profiles of membrane proteins from human breast cancer cell and tissue specimens. First, the membrane proteins of cell lines or tissues were isolated using a modified detergent-based protein extraction method. The purity of the obtained membrane proteins was confirmed by Western blotting using anti-Na^+^/K^+^ ATPase-α1 and anti-γ-tubulin antibodies (Figure 1A). In Figure 1A, Na^+^/K^+^ ATPase-α1 was only present in the detergent phase of Bcap37 cell and tumor tissue samples, but not in the liquid phase, which contained cytoplasmic proteins. Therefore, the detergent phase of cell and tissue samples contained mostly membrane proteins. This result indicated that the detergent-based method was capable of perfectly isolating membrane proteins from total proteins to make membrane proteins suitable for the following analysis.

GC and Sephadex G10 resins were introduced as purification agents to make N-glycans suitable for N-glycan profiling. The GC resin is able to remove salts, detergent and polypeptide contaminants from the crude N-glycans sample while excessive APTS could be removed by Sephadex G10 resin. In this study, the GC resin purification step was further optimized. Gradient elution was adopted to purify glycans in which elution buffers I, II, III were successively used twice and elution IV thrice. Meanwhile eluotropic centrifugation was adjusted to keep the flow rate at 0.5-1.0 mL/min. Results indicated that the quality of N-glycan profiling was remarkably improved, especially in the peak numbers and relative heights of glycans in comparison with that of the isocratic elution method (25% ACN + 0.075% TFA) [31] and purification-free method (Figure S2).

**Figure 2:**
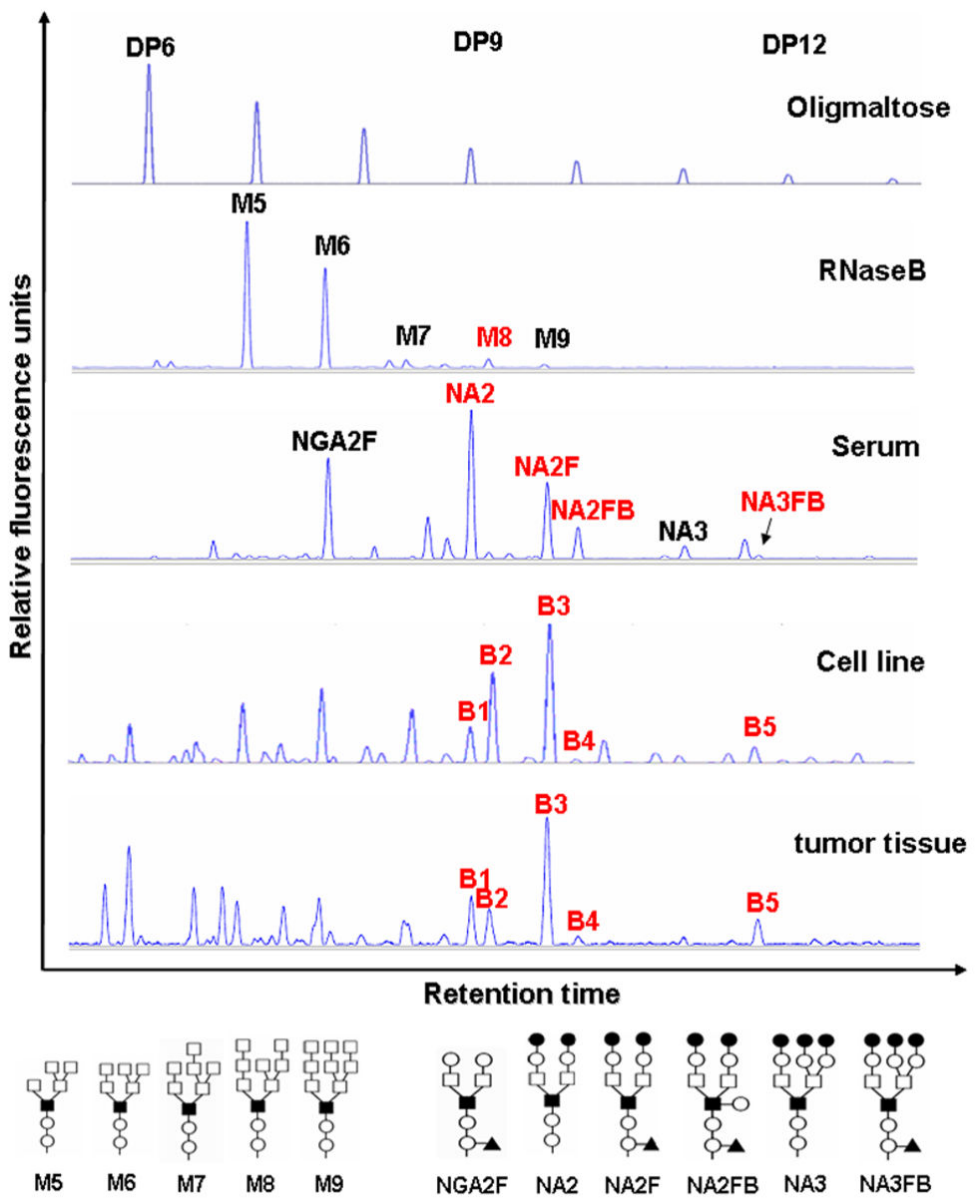
The representative N-glycan profiles of membrane proteins from human breast cancer cell line and tissue. The desialylated N-glycan profiles of membrane proteins from cell lines (n=8) and tumor tissues (n=100). Oligomaltose is used as a sugar mass reference. The number of glucose units (degree of polymerization, DP) in these structures is indicated. N-glycan profiles from RNaseB and serum were used as N-glycan profile controls. RNaseB contained high mannose from M5 to M9. Serum contained various complex N-glycans and the most abundant glycans detected in serum are marked. The vertical axis represents the glycan intensity of the peaks as a percent of the relative fluorescence level. The X-axis represents the retention time of the peaks. The N-glycan structures of the corresponding peaks are shown below the panels. NGA2F is an agalacto core-α-1, 6-fucosylated biantennary glycan; NA2 is a bigalacto biantennary glycan; NA2F is a bigalacto core-α-1, 6-fucosylated biantennary glycan; NA2FB is a bigalacto core-α-1, 6-fucosylated bisecting biantennary glycan; NA3 is tri-antennary; NA3FB is a core-α-1, 6-fucosylated triantennary glycan. The symbols used in the structural formulas are as follows: (○) β-linked N-acetylglucosamine; (●) β-linked galactose; (□) α-linked mannose; (■) β-linked mannose; (▲) α-1, 6-linked fucose.

Using this modified method, we first compared the N-glycan profiles of cytosolic and membrane proteins from breast cancer cell line and tissue specimens. As shown in Figure 1B, the N-glycan profiling of membrane proteins was dramatically different from that of cytosolic proteins. Although more than 10 peaks were detected before DP8, most signal peaks (e.g., B1 to B5 as shown in Figure 2) were located from DP8 to DP12 in N-glycan profiling of membrane proteins. These five peaks (peak B1 to B5) were studied in the following studies because (1) only the peaks falling the range of DP8-DP12 are membrane-protein-specific; (2) all of them could be found in all of breast cancer cell lines and tissues and also showed a high fluorescent intensity. These results indicated that the modified DSA-FACE technique could help with obtaining well separated membrane proteins from cell line and tissue samples in the sample preparation step, and to obtain better N-glycan profiling (Figure 2).

### Structural analysis of N-glycans

**Figure 3:**
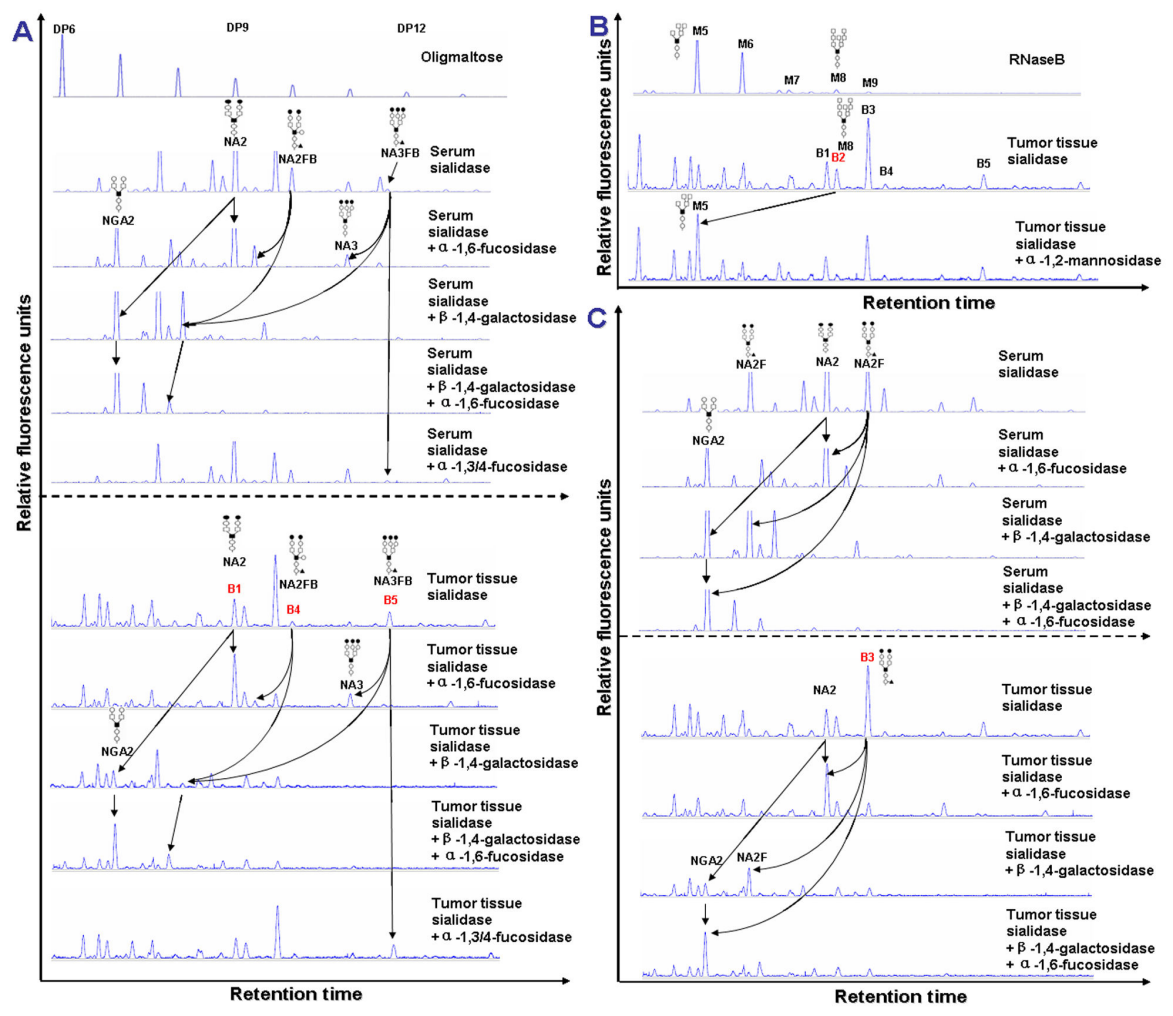
The exoglycosidase sequencing of the membrane protein N-glycans from tumor tissues. (A) Exoglycosidase sequencing of N-glycans of membrane proteins from breast cancer tissue (lower panel) and glycoproteins from healthy human serum as reference (upper panel) to verify the structures of peaks B1, B4 and B5. The structures of peaks B2 and B3 are confirmed in (B) and (C), respectively. The total N-glycans were treated with single or combined exoglycosidase arrays as indicated in context. The arrow lines indicate the changes in glycan peaks that underwent glycosidase digestion. The nomenclature of N-glycans and symbolic representations correspond to those in Figure 2.

RNaseB, a glycoprotein, carries multiple types of glycans, most of which are high-mannose N-glycans. The structures of these N-glycans such as M5-M9 (RNaseB was digested by PNGase F) have been characterized [32,33]. While, the structures of NGA2F to NA3FB were also revealed in the glycoproteins from human serum [34]. In this study, in order to determine the structures of the N-glycans from cell or tissue samples, we compared their N-glycan profiles with that of RNaseB and serum whose N-glycan profiles were already known (Figure 2). Peaks B1, B4 and B5 migrated at the same rates as NA2, NA2FB and NA3FB, respectively, in the corresponding human serum N-glycan profile. The N-glycan structures of these peaks were further studied by exoglycosidase digestion (Figure 3A, lower panel). Two, two and three galactoses could be removed from N-glycans responding to peaks B1, B4 and B5, respectively, by β-1, 4-galactosidase. When β-1, 4-galactosidase was combined with α-1, 6-fucosidase, one extra fucose residue was taken off from peaks B4 and B5. Nevertheless peaks B4 and B5 could not be digested by α-1, 3/4-fucosidase. This suggested that peaks B4 and B5 were the substrates of α-1, 6-fucosidase but not α-1, 3/4-fucosidase. These results indicated that peak B5 was a core-α-1, 6-fucosylated triantennary glycan (NA3FB). Moreover, the motion trails of peaks B1 and B4 that had undergone the digestion of β-1, 4-galactosidase or α-1, 6-fucosidase, or their mixture, were consistent with those of the reference glycans NA2 and NA2FB in human serum (Figure 3A, upper panel). In addition, we also found that peaks B2 and B3 migrated at the same site as M8 and NA2F or M9, respectively. Thus, the structures of peaks B2 and B3 were confirmed as M8 and NA2, respectively, by α-1, 2-mannosidase and a series of exoglycosidases as mentioned above (Figure 3B and 3C). The structures of the N-glycans in tumor cell lines were also found to be consistent with that in tumor tissues (Figure S3). We therefore assigned the peaks B1, B2, B3, B4 and B5 in the N-glycan profiles from breast cancer cell lines or tissues as NA2, M8, NA2F, NA2FB and NA3FB, respectively.

### Alteration of the cell surface-specific N-glycans from breast cancer cell lines

**Figure 4:**
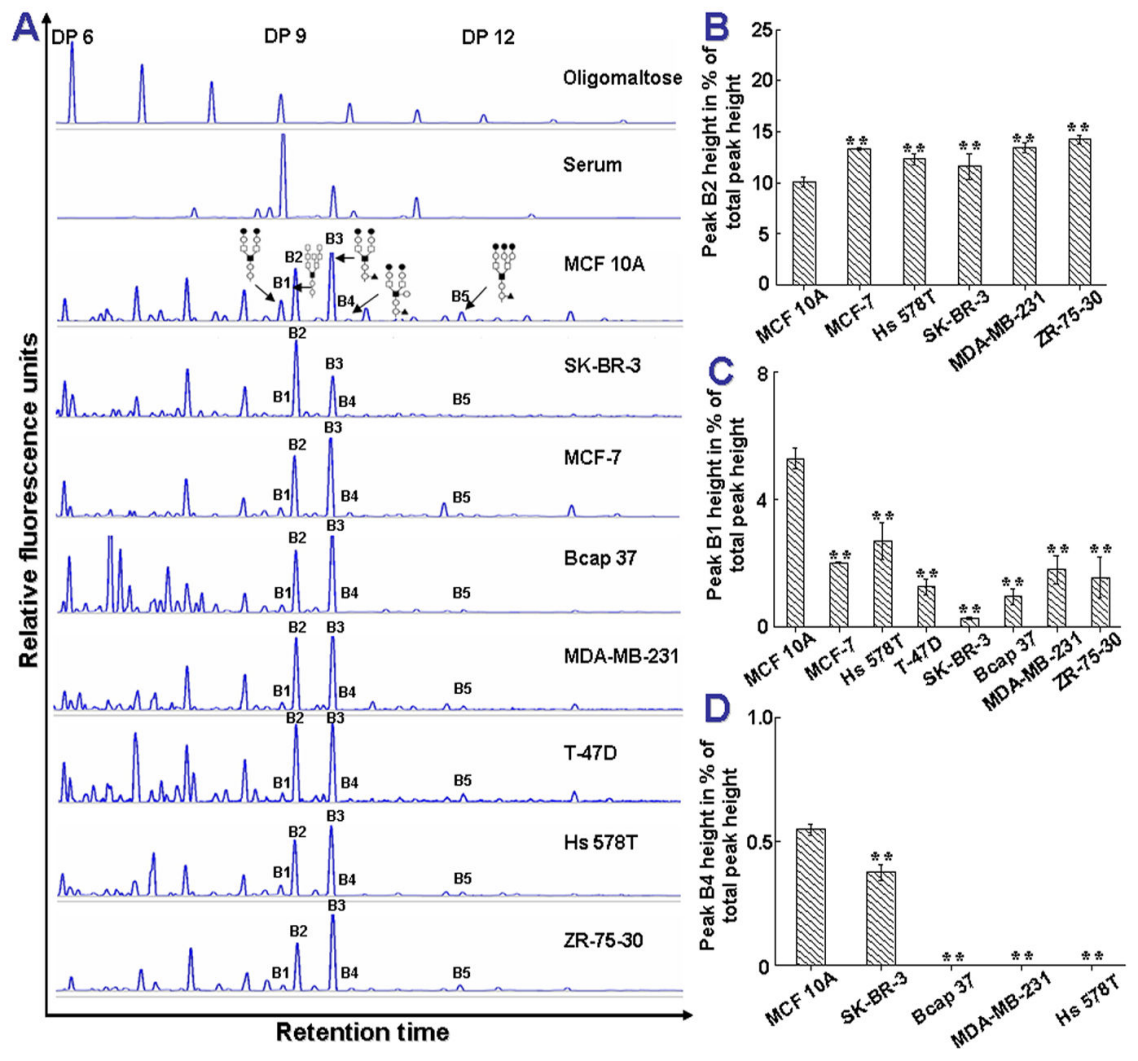
The significantly changed N-glycans of the membrane proteins of human breast cancer cell lines. (A) The representative N-glycan profiles of membrane proteins from human breast cancer cell lines (n=7). MCF 10A: from breast fibrocystic disease as a non-tumorigenic epithelial cell line; SK-BR-3, MCF-7, Bcap 37, MDA-MB-231, T-47D, Hs 578T and ZR-75-30: cancer cell lines; Serum: from healthy humans. (B–D) The statistically significant difference in N-glycan peaks B1, B2, B4 and B5 between MCF 10A and various cancer cell lines. Peak B2 is significantly increased in cancer cell lines compared with MCF 10A. On the contrary, peaks B1 and B4 are dramatically decreased in cancer cell lines. Particularly, peak B4 is completely lost in Bcap 37, MDA-MB-231 and Hs 578T cell lines. The percentages of each specific peak height in the total peak heights are expressed as mean ± SD. Asterisks indicate statistically significant differences between the various cancer cell lines and the MCF 10A cell line (* *p* < 0.05, ** *p* < 0.01). Five major glycan peaks B1-B5 in normal control and breast cancer groups were detected. Experiments were repeated three times.

The N-glycan profiles of membrane proteins from cell lines were obtained and analyzed using the above modified method. Figure 4A displayed the N-glycan profiles of a non-tumorigenic epithelial cell line MCF 10A and 7 breast cancer cell lines (SK-BR-3, MCF-7, Bcap37, MDA-MB-231, T-47D, Hs-578T and ZR-75-30). We compared the corresponding peaks in N-glycan profiles of MCF 10A and each tumor cell line. Results showed that peaks B1, B2 and B4 were significantly altered in most of the tumor cell lines (Figure 4B, C and D). Peak B2, which represented high mannose N-glycan (M8), was significantly increased (Figure 4B) but peaks B1 (NA2) and B4 (NA2FB) were significantly decreased in cancer cell lines in comparison with MCF 10A. In particular, peak B4 (NA2FB) was completely lost in Bcap37, MDA-MB-231 and Hs-578T cells (Figure 4C and D).

### Alteration of cell surface-specific N-glycans from clinical breast cancer tissues

Based on the results of the cell lines, 100 pairs of N-glycan profiles of membrane proteins from matched normal and malignant tissues were isolated from breast cancer patients. The N-glycans were represented by peaks B1-B5 which corresponded to the cell lines.

The clinicopathological characteristics of breast cancer specimens from the 100 patients are presented in Table 1. Specimens were characterized according to the patients’ age, lymph node metastasis, hormonal and HER-2 receptor status and tumor grade according to TNM stages.

**Table 1:** Clinical Characteristics of the Study Population.

Characteristics	Category	Cases	Percentage (%)
Age	<=40	23	23.0
	41-50	32	32.0
	51-60	31	31.0
	>60	14	14.0
	Mean	49.8	
Metastasis	LN ^b)^	47	58.0
Hormone receptor	ER ^c)^ (+)	46	68.7
	ER ^c)^ (-)	21	31.3
	ND ^a)^	33	
	PR ^d)^ (+)	41	61.2
	PR ^d)^ (-)	26	38.8
	ND ^a)^	33	
Her-2	Her-2 (3+)	5	8.7
	Her-2 (2+)	13	20.0
	Her-2 (+/-)	47	72.3
	ND ^a)^	35	
TNM stage	I	11	13.9
	II	41	51.9
	III-IV	27	34.2
	ND ^a)^	21	

^a)^ ND, not determined; ^b)^ LN, lymph node; ^c)^ ER, estrogen receptor; ^d)^ PR, progesterone receptor.

**Figure 5:**
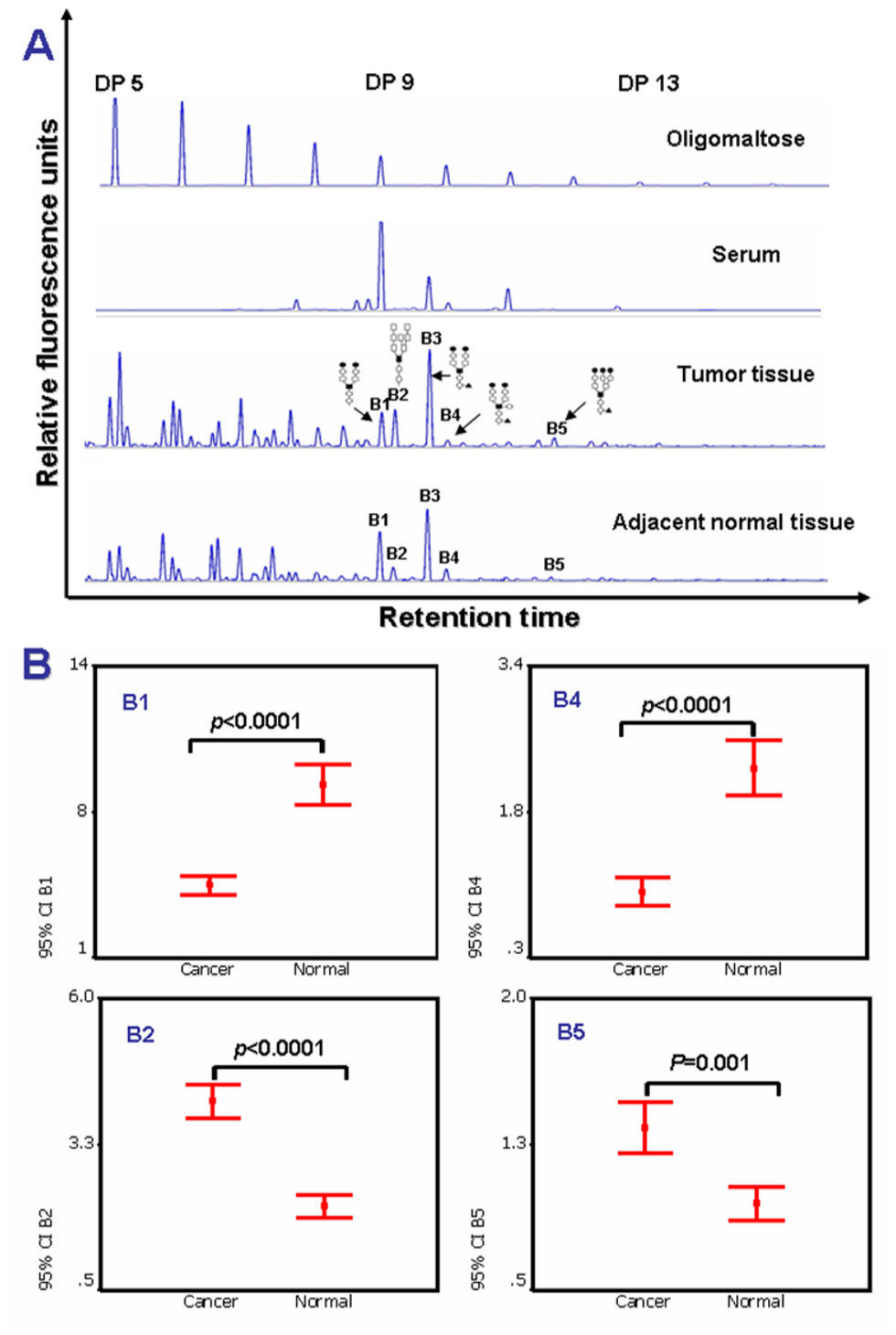
The significantly changed N-glycans in breast cancer tissues compared to adjacent normal tissues. (A) The typical cell surface-specific N-glycan profiles from breast cancer tissue (n=100) and adjacent normal tissue (n=100). (B) The statistically significant differences of N-glycan peaks B1, B2, B4 and B5 between breast cancer and adjacent normal tissues. The statistically significant differences between groups are indicated as *p* value. The structures of the N-glycan peaks B1, B2, B4 and B5 are shown in the Figure 2.

Paired and unpaired *t*-tests were used to compare the two tissue groups and the intra- and inter-assay coefficients of variations (CVs) of the glycan analysis were less than 5%. Results showed that the N-glycan profiles of breast cancer tissues were significantly distinguished from that of corresponding adjacent tissues (Figure 5A). Peaks B1 (NA2) and B4 (NA2FB) were significantly decreased in the tumor group (*p*<0.0001) compared with the corresponding normal tissue group, which was consistent with those in the cancer cell lines (Figures 4B and 5B). Furthermore, peaks B2 (M8) and B5 (NA3FB) were significantly increased in the tumor group (*p*<0.0001 and *p*=0.001) compared to the corresponding normal tissue group (Figure 5B). These results indicated that NA2, NA2FB, M8 and NA3FB were dramatically altered on the surface of breast cancer cells.

### Alteration of specific N-glycans in breast cancer patients with different ages and clinical stages

**Figure 6:**
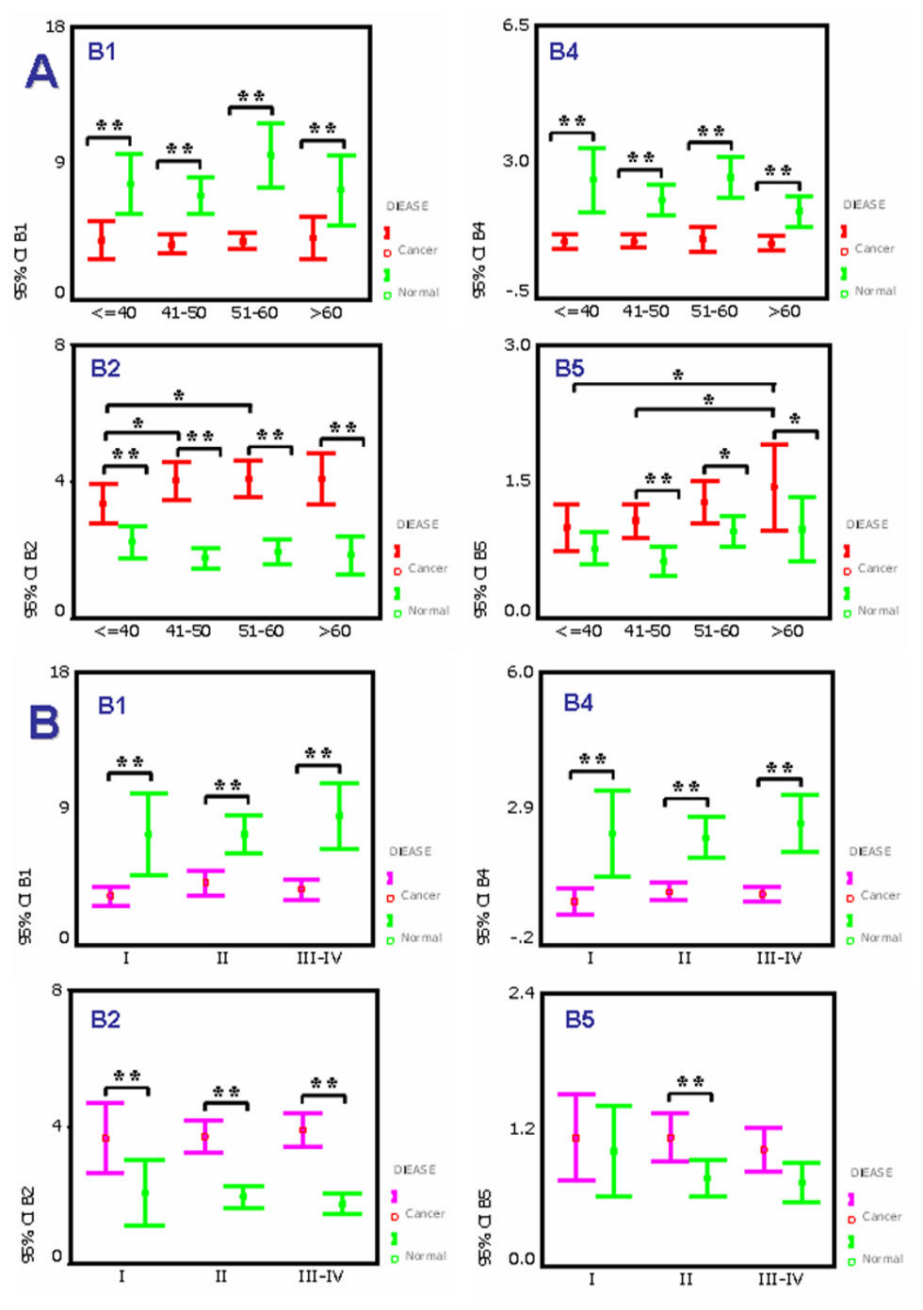
The significantly changed N-glycans in breast cancer tissues according to age and clinical stage. (A) The correlation of cell surface specific N-glycans with four age groups. (B) The correlation of cell-surface specific N-glycans with clinical stages. Statistically significant differences between groups is indicated by *p* value (* *p* < 0.05, ** *p* < 0.01). The structures of N-glycan peaks B1, B2, B4 and B5 are shown in Figure 2.

The concentrations of biantennary glycans (NA2 and NA2FB), a triantennary glycan (NA3FB) and a high-mannose glycan (M8) in breast cancer patients with different ages and clinical stages were compared to study the correlation between these N-glycans and patient ages and clinical stages (Table 1). Both peaks B1 (NA2) and B4 (NA2FB) from tumor tissue specimens were significantly decreased in all ages (Figure 6A) and clinical stage groups (Figure 6B) compared with corresponding normal tissue specimens. Peak B2 (M8) was not only significantly increased in all age and stage groups but also significantly higher in tumors from patients >40 years of age. These results suggested that the alterations of peaks B1 (NA2), B2 (M8) and B4 (NA2FB) are part of the oncogenic process of breast cancer. It is worth noting that the alteration of peak B5 (NA3FB) trended higher with age >40 and with > stage I tumors. Although peak B5 (NA3FB) did not have a statistically significant difference (*p*=0.057) relative to normal tissues in stages III and IV tumors as compared to stage II tumors, this is probably due to the limited number of specimens analyzed. Based on this observation, we speculate that NA3FB may be associated with malignant progression of breast cancer.

### Correlation between specific N-glycans and clinical pathologic features

The correlation between N-glycans and hormone receptors, the Her-2 receptor and lymph node metastasis (LN) status was analyzed in breast cancer patients using Pearson correlation. However, the results showed that NA2, NA2FB, M8 and NA3FB were not closely correlated with the clinical pathologic characteristics of the tumors (Table S1).

## Discussion

Glycomics has grown to be a research hotspot due to the awareness of the important biological functions of glycosylation and rapid development of glycan researching approaches. In the past decade, DSA-FACE, as an N-glycan profiling detection technique, has drawn much attention due to its advantages such as high sensitivity, high throughput and quick analysis. It has been widely accepted as a biomarker screening approach in cancer and other disease research. For instance, DSA-FACE has been applied to identify serum protein biomarkers for liver fibrosis (GlycoFibroTest) [20], hepatic cirrhosis (GlycoCirrohTest) [35], hepatocellular cancer (GlycoHCCTest) [34], Alzheimer’s disease [17] and colorectal cancer (CRCglycoA/B) [27]. However, to our knowledge, there has been no previous report on membrane protein N-glycan profiling from tissues or cell lines obtained with DSA-FACE, which could be a limitation of its further use in glycomics. In this study, we established a DSA-FACE-based method that was optimized for the profiling of cell surface-specific N-glycans from human breast cancer cell lines and tissues.

N-glycan profiles of membrane proteins from human breast cancer cells and tissues were obtained with the modified DSA-FACE method. It was found that the relative levels of NA2 (peak B1) and NA2FB (peak B4) in breast cancer groups were dramatically decreased (*p*<0.0001) compared to corresponding control groups. This indicated that NA2 and NA2FB were the dramatically changed functional N-glycans on breast cancer cell surfaces. Some previous studies reported that NA2 was less abundant in hepatocellular cancer (HCC) patients with hepatitis C virus (HCV) than in healthy humans [36] and NA2FB was found to be significantly decreased in HCC patients with HBV in comparison to those with cirrhosis [34]. Therefore, NA2 and NA2FB were closely associated with HCC and probably breast cancer as well. It is well known that glycans are not antigenic by themselves but are able to be antigenic if they bond to large carrier molecules such as proteins. Glycosylation contributes to antigen presentation and the product-glycoprotein can be antigenic to elicit various anti-carbohydrate antibodies [37]. Cancer cells are the altered-self cells that have their own antigens. The immune system is able to recognize and destroy cancer cells via recognizing the tumor-specific antigens on cell surfaces. However, cancer cells can successfully evade immunosurveillance via some mechanisms include shedding and/or modulating antigens. From this point of view, a decrease in the membrane glycoproteins glycans enables cancer cells to mask or lose antigenic epitopes, which consequentially leads to the immunologic escape of tumor cells. Cormier et al. [38] found the expression of melanoma-associated antigens (MAAs; a glycoprotein on cell membranes) was decreased to 50% to allow melanoma cells escape the recognition by HLA class I-restricted cytotoxic T cells. In this study, the decreased cell surface N-glycans NA2 and NA2FB may also be related to the immunologic escape of breast cancer cells. Further study on these altered N-glycans may provide a novel route to demonstrate the immunologic escape mechanism of breast cancer cells.

In addition, a branching α-1, 6-fucosylated triantennary glycan, NA3FB (peak B5), was found to be significantly increased in breast cancer patients. Also, this variation trend for peak B5 was exactly consistent with that in breast cancer serum (our unpublished data). Nakagawa et al. [39] also found that NA3FB was increased in α-fetoproteins (AFPs) from HCC by detecting the N-glycan structures of AFPs from cell lines, serum and ascites fluids from HCC patients. These results are consistent with our findings in breast cancer. The same mechanism seems to regulate NA3FB in hepatocellular and breast cancer cells and the change in NA3FB may be involved in tumor progression. Lebrilla et al. [40] proposed that the elevation of the high-mannose glycans probably was a common phenomenon in breast cancer because they found the level of M9 significantly increased in serum samples from breast cancer in mice and humans. In this study, another high-mannose, M8, was also found to be elevated on the cell surface of breast cancer cells compared with normal cells. Furthermore, M8 was not only significantly increased in the patients of different ages and TNM stages but also gradually increased with patient age until 60-years-old. To date, several tumor-specific carbohydrates were increased by different degrees and have been found be associated with breast cancer and have been used in tumor therapy as targeted antigens, such as Tn antigen, sialyl Lewis X antigen, MUC1 glycoprotein, Her-2/Neu glycoprotein and CEA [41]. Consequently, the upregulated N-glycans NA3FB and M8 in this study could serve as new potential breast cancer-specific carbohydrate antigens. In the future, a new drug targeting NA3FB or M8 holds the promise of being more selective for cancer cells and harming fewer normal cells, therefore, reducing side effects and improving quality of life.

**Figure 7:**
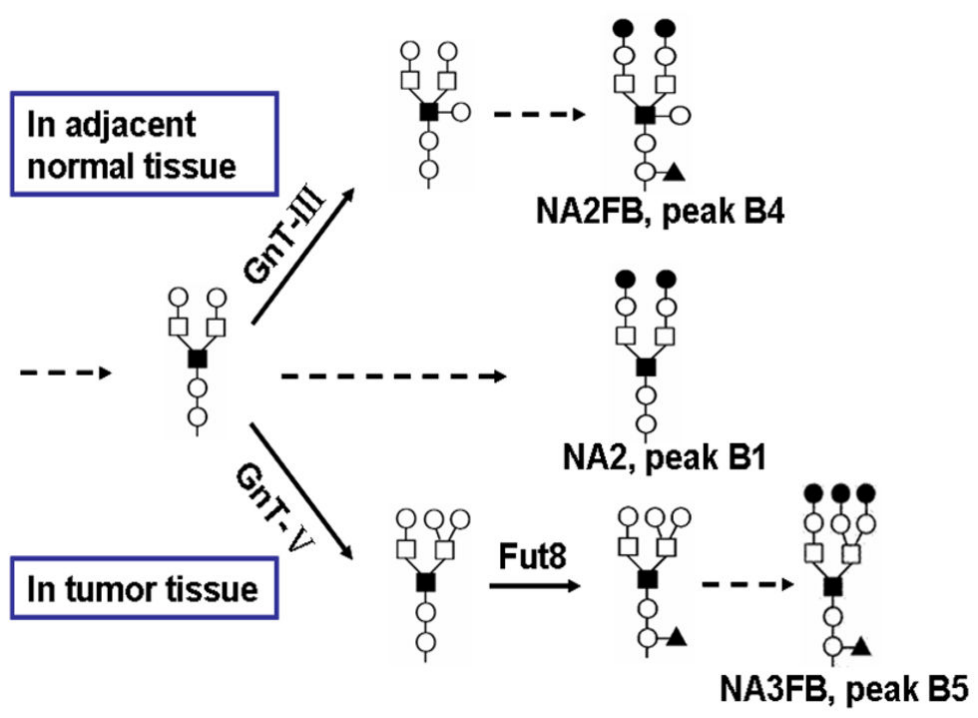
The schematic presentation of changes in N-linked glycans in breast cancer. The increased concentration of NA3FB (peak B5) and decreased concentration of NA2 (peak B1) and NA2FB (peak B4) in breast cancer tissues could be attributed to the increased activity of GnT-V, which competes for substrate with GnT-III and is associated with the consequential elevated level of branched N-glycans.

Based on the N-glycan structure analysis, we inferred that the alternations of biantennary glycan NA2, bisecting biantennary glycan NA2FB and triantennary glycan NA3FB could be attributed to the changed expression of two glycosyltransferases GnT-III and GnT-V. Pawelek et al. reported that the expression of GnT-V was strongly up-regulated in breast cancer [42]. Thus, according this study, GnT-V which generates triantennary glycan NA3FB could compete as a substrate with GnT-III which produces bisecting GlcNAc (NA2FB). This leads to an increased abundance of NA3FB and decreased abundance of NA2 and NA2FB during tumor development in breast cancer patients (Figure 7). Furthermore, it has been reported that the increased triantennary glycans could result in aberrant N-glycosylation of her-2 and/or the erbB family receptors in the oncogenesis and progression of breast cancer [43]. This aberrant alteration of the receptor family members on cell surfaces can regulate their endocytosis and lead to the altered binding of ligands (e.g., EGF), which consequently affects the cancer signaling pathways mediated by these receptors. From this point of view, the significantly altered cell surface-specific N-glycans, especially those from critical receptors on cell surfaces regulated by various glycosyltransferases, are potential participants in tumor progression in breast cancer.

In conclusion, this study analyzed the cell surface-specific N-glycans from cell line and tissue specimens of breast cancer patients for the first time using a modified DSA-FACE technique. It was found that the N-glycans NA2 and NA2FB were significantly decreased in 100 human breast cancer tissue samples compared with corresponding adjacent normal tissue samples, while M8 and NA3FB were dramatically increased. Moreover, the alterations of these specific changed N-glycans were also found through the oncogenesis and progression of breast cancer by a correlation analysis between the N-glycan profiles of breast cancer tissue specimens and patient ages and tumor clinical stages. These dramatically changed N-glycans on tumor cell surfaces are not only promising drug targets for breast cancer therapy but they also help with understanding the mechanism of tumor cell immunologic escape in breast cancer.

## Supporting Information

Figure S1 **The workflow scheme of high-throughput membrane protein N-glycan preparation and analysis using a DNA sequencer.**
(TIF)

Figure S2 **The N-glycan profiles of breast cancer cell lines or tissues with different purification methods.** (A) The N-glycan profiling of 160 μg membrane proteins from breast cancer cell line Bcap 37 without purification (upper profiling), with relatively simple purification (middle profiling) and optimal purification (lower profiling). (B) The N-glycan profiling of 160 μg membrane proteins from breast cancer tissue without purification (upper profiling), with relatively simple purification (middle profiling) and optimal purification (lower profiling).
(TIF)

Figure S3 **The exoglycosidase sequencing of N-glycans of membrane proteins from breast cancer cell line T47D.** Arrow lines indicate the changes of glycan peaks due to glycosidase digestion. The nomenclature of N-glycans and symbolic representations correspond to that in Figure 2.
(TIF)

Table S1 **The pearson correlation coefficient between specific N-glycans and clinical pathological features.**
(DOC)
