# Supplementary figures and images for: Cell Surface-Specific N-Glycan Profiling in Breast Cancer
Source: PLoS One. 2013 Aug 23;8(8):e72704. doi: 10.1371/journal.pone.0072704 (PMC3751845; doi:10.1371/journal.pone.0072704)

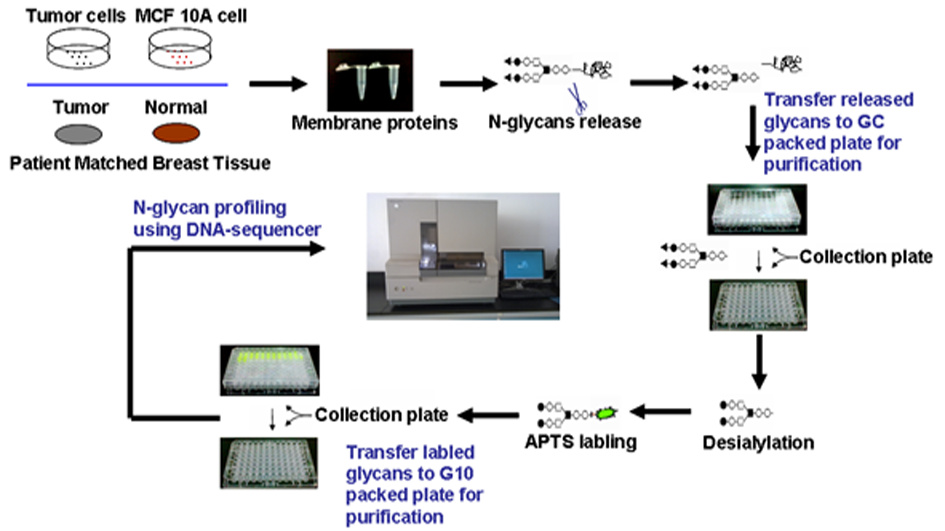

Supplement: Figure S1 — The workflow scheme of high-throughput membrane protein N-glycan preparation and analysis using a DNA sequencer. (TIF) [file pone.0072704.s001.tif]

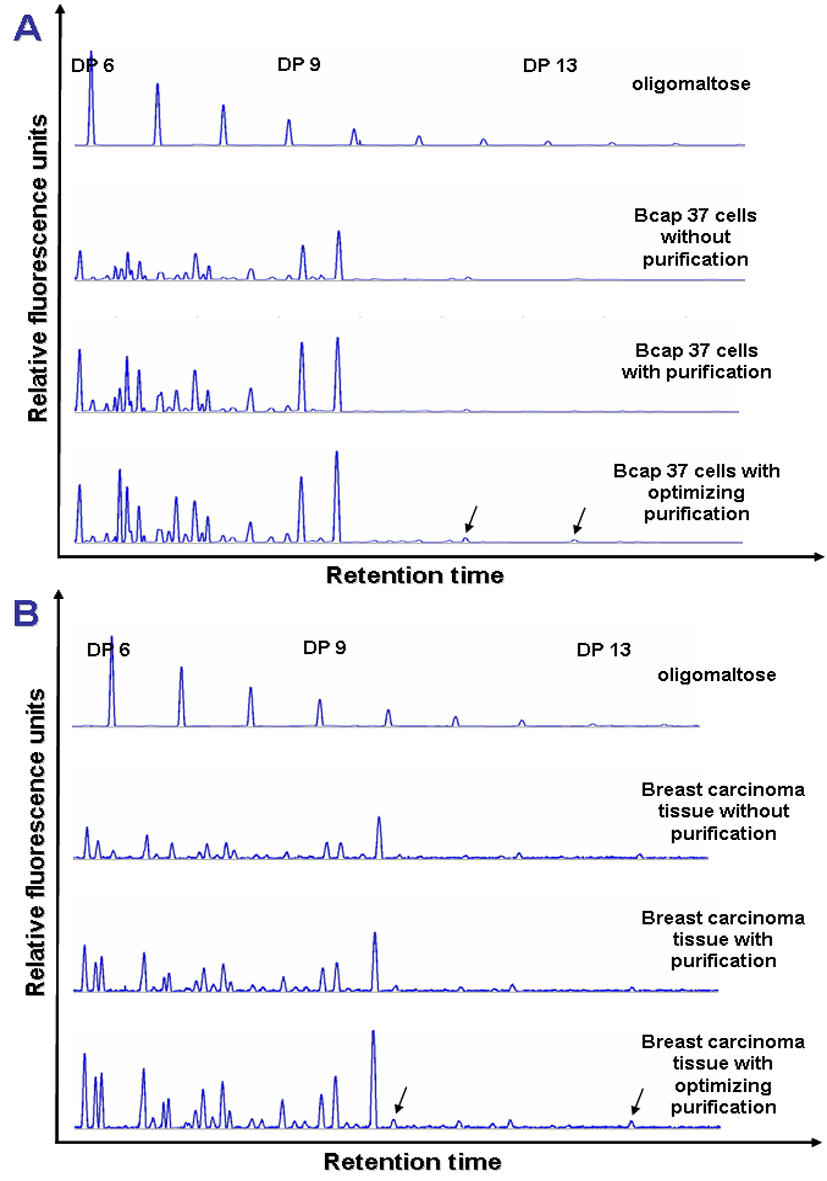

Supplement: Figure S2 — The N-glycan profiles of breast cancer cell lines or tissues with different purification methods. (A) The N-glycan profiling of 160 μg membrane proteins from breast cancer cell line Bcap 37 without purification (upper profiling), with relatively simple purification (middle profiling) and optimal purification (lower profiling). (B) The N-glycan profiling of 160 μg membrane proteins from breast cancer tissue without purification (upper profiling), with relatively simple purification (middle profiling) and optimal purification (lower profiling). (TIF) [file pone.0072704.s002.tif]

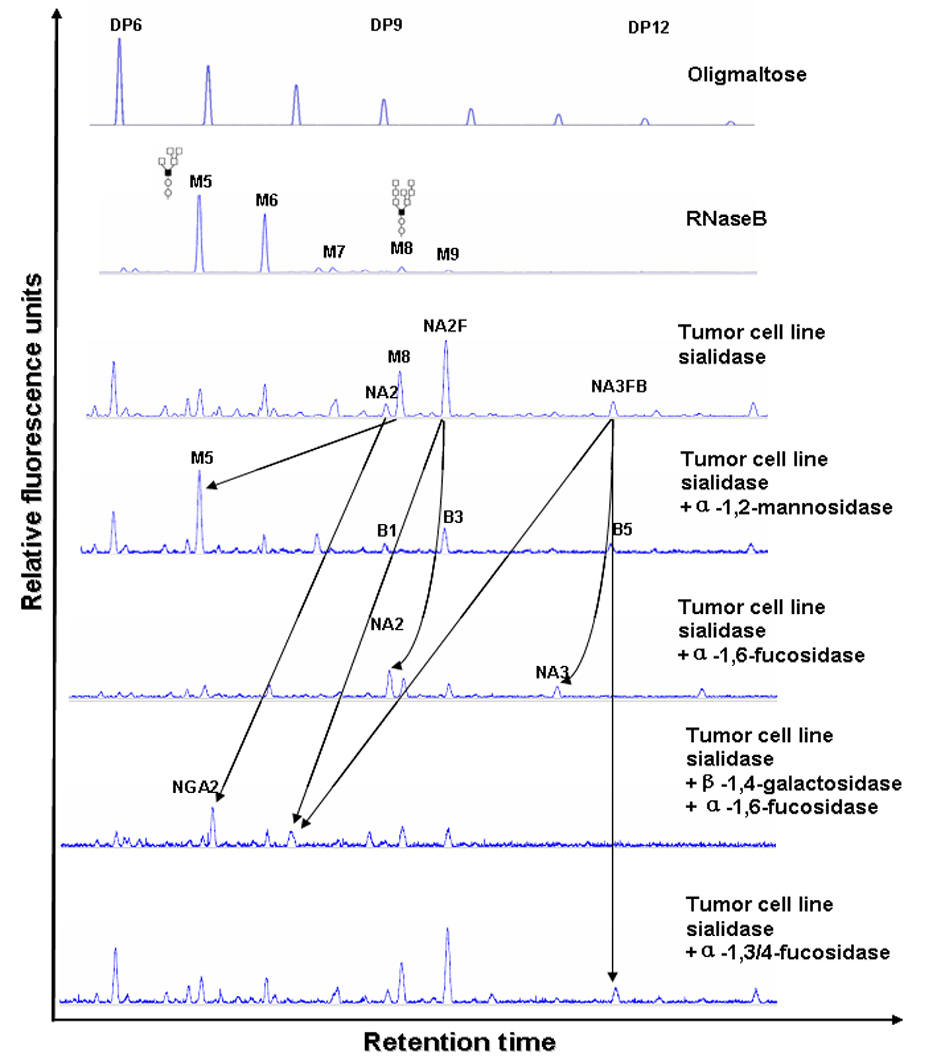

Supplement: Figure S3 — The exoglycosidase sequencing of N-glycans of membrane proteins from breast cancer cell line T47D. Arrow lines indicate the changes of glycan peaks due to glycosidase digestion. The nomenclature of N-glycans and symbolic representations correspond to that in Figure 2. (TIF) [file pone.0072704.s003.tif]
