# Supplementary material for: Cell Surface-Specific N-Glycan Profiling in Breast Cancer
Source: PLoS One. 2013 Aug 23;8(8):e72704. doi: 10.1371/journal.pone.0072704 (PMC3751845; doi:10.1371/journal.pone.0072704)
Supplement: Table S1 — The pearson correlation coefficient between specific N-glycans and clinical pathological features. (DOC) [file pone.0072704.s004.doc]

Table S1. The pearson correlation coefficient between specific N-glycans and clinical pathological features

|  |  | ER status | PR status | HER-2 status | LN status |
| --- | --- | --- | --- | --- | --- |
| Peak B1 | Pearson Correlation | -0.053 | -0.007 | -0.072 | 0.052 |
|  | Sig. (2-tailed) | 0.670 | 0.955 | 0.570 | 0.655 |
| Peak B2 | Pearson Correlation | 0.009 | -0.024 | 0.126 | -0.082 |
|  | Sig. (2-tailed) | 0.941 | 0.845 | 0.318 | 0.483 |
| Peak B4 | Pearson Correlation | 0.126 | 0.172 | -0.199 | -0.097 |
|  | Sig. (2-tailed) | 0.311 | 0.164 | 0.112 | 0.404 |
| Peak B5 | Pearson Correlation | -0.190 | -0.028 | 0.142 | 0.032 |
|  | Sig. (2-tailed) | 0.124 | 0.820 | 0.259 | 0.784 |
